# Supplementary material for: Structural basis of drug recognition by human MATE1 transporter
Source: Nat Commun. 2025 Oct 27;16:9444. doi: 10.1038/s41467-025-64490-z (PMC12559748; doi:10.1038/s41467-025-64490-z)
Supplement: Supplementary file 1 — Supplementary Information [file 41467_2025_64490_MOESM1_ESM.pdf]

Supplementary Information for

Structural basis of drug recognition by human MATE1 transporter

Ksenija Romane[1], Giulia Peteani[2,3], Somnath Mukherjee[4], Julia Kowal[1], Lorenzo Rossi[1], Jingkai Hou[4], Anthony A. Kossiakoff[4], Thomas Lemmin[2], Kaspar P. Locher[1]

[1]Institute of Molecular Biology and Biophysics, ETH Zürich, 8093 Zürich, Switzerland

[2]Institute of Biochemistry and Molecular Medicine, Universität Bern, 3012 Bern, Switzerland

[3]Graduate School for Cellular and Biomedical Sciences (GCB), University of Bern, 3012 Bern, Switzerland

[4]Department of Biochemistry and Molecular Biology, The University of Chicago, Chicago, IL, USA

This file contains:

Supplementary Figures 1-13
Supplementary Tables 1,2

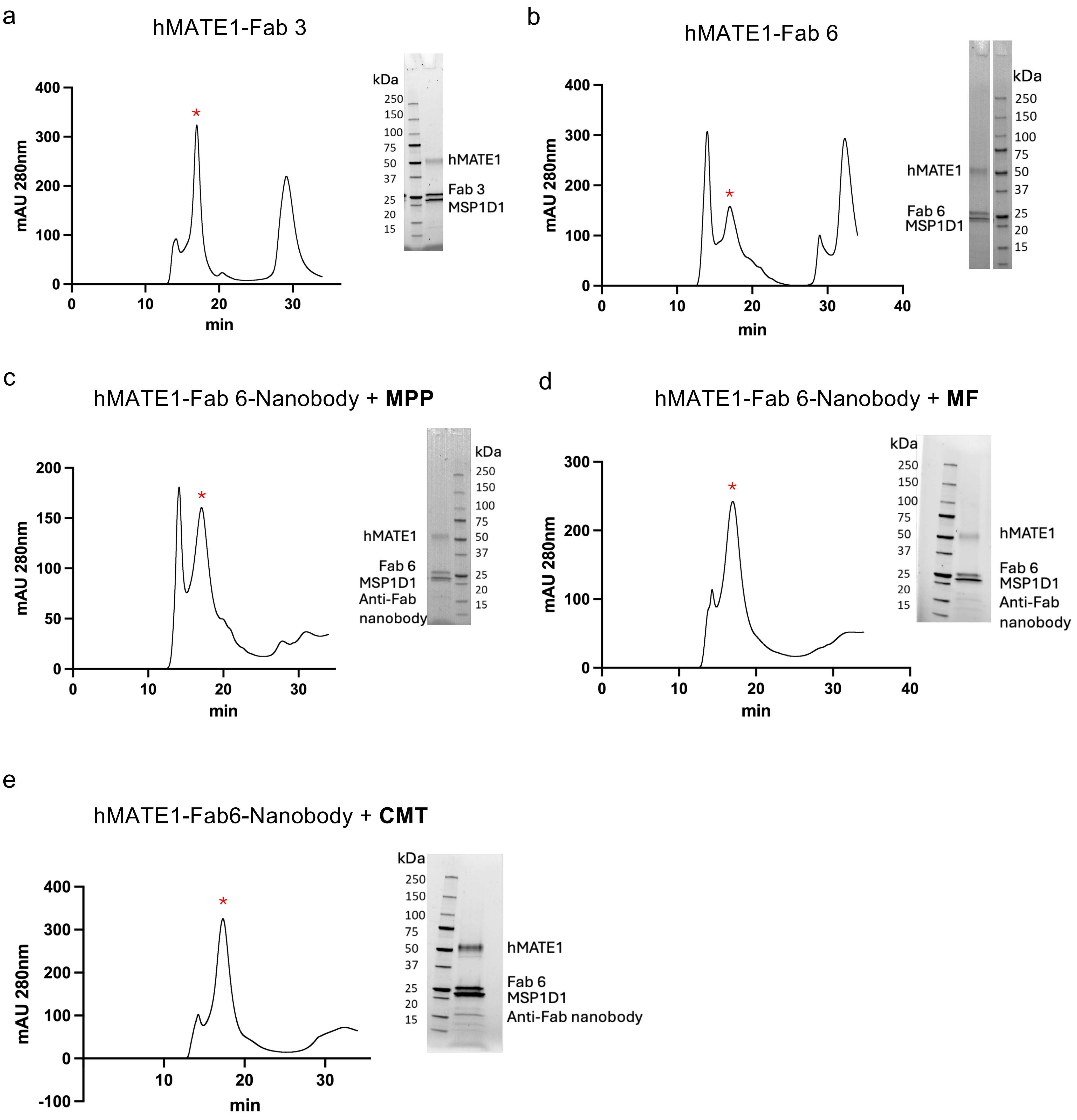

Supplementary Fig.1. Sample preparation of hMATE1 for cryo-EM studies. **a** Size exclusion chromatography (SEC) of hMATE1-Fab 3 complex. **b, c, d, e** SEC of hMATE1-Fab 6 complexes. The fraction used for grid preparation is shown with asterisc and on SDS-PAGE gel. To each fraction either no substrate or substrate (in bold) was added before freezing on grids.

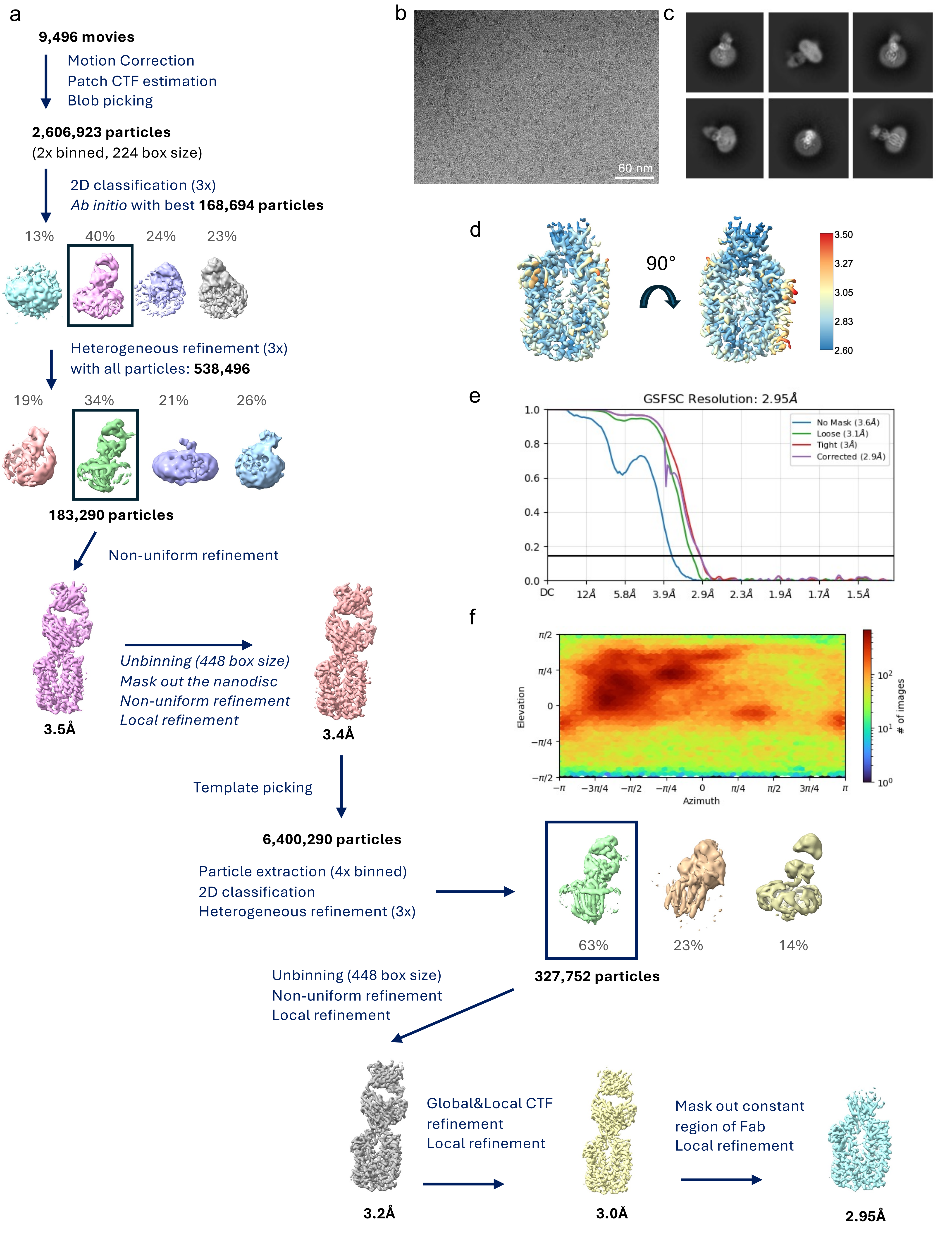

Supplementary Fig. 2. Cryo-EM analysis of hMATE1 in a complex with MATE1_Fab 6. **a** Flowchart presenting the pipeline of data processing in CryoSparc. **b** Representative micrograph of imaged sample. **c** Selected 2D classes. **d** Local resolution estimates of the final EM density map. **e** FSC curves of the final EM density maps. **f** Angular distribution of particles used in the final reconstruction.

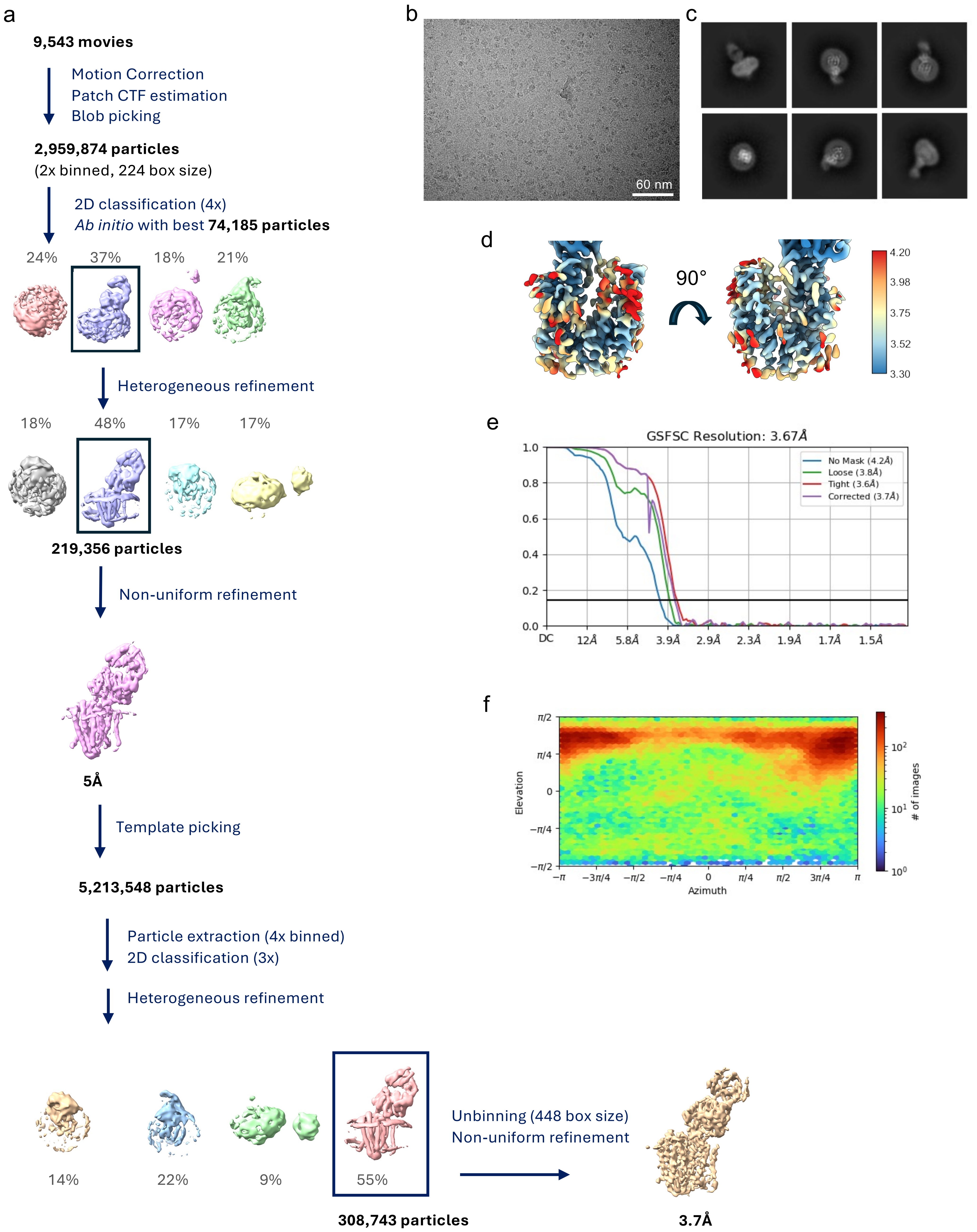

Supplementary Fig. 3. Cryo-EM analysis of hMATE1 in a complex with MATE1_Fab 3. **a** Flowchart presenting the pipeline of data processing in CryoSparc. **b** Representative micrograph of imaged sample. **c** Selected 2D classes. **d** Local resolution estimates of the final EM density map. **e** FSC curves of the final EM density maps. **f** Angular distribution of particles used in the final reconstruction.

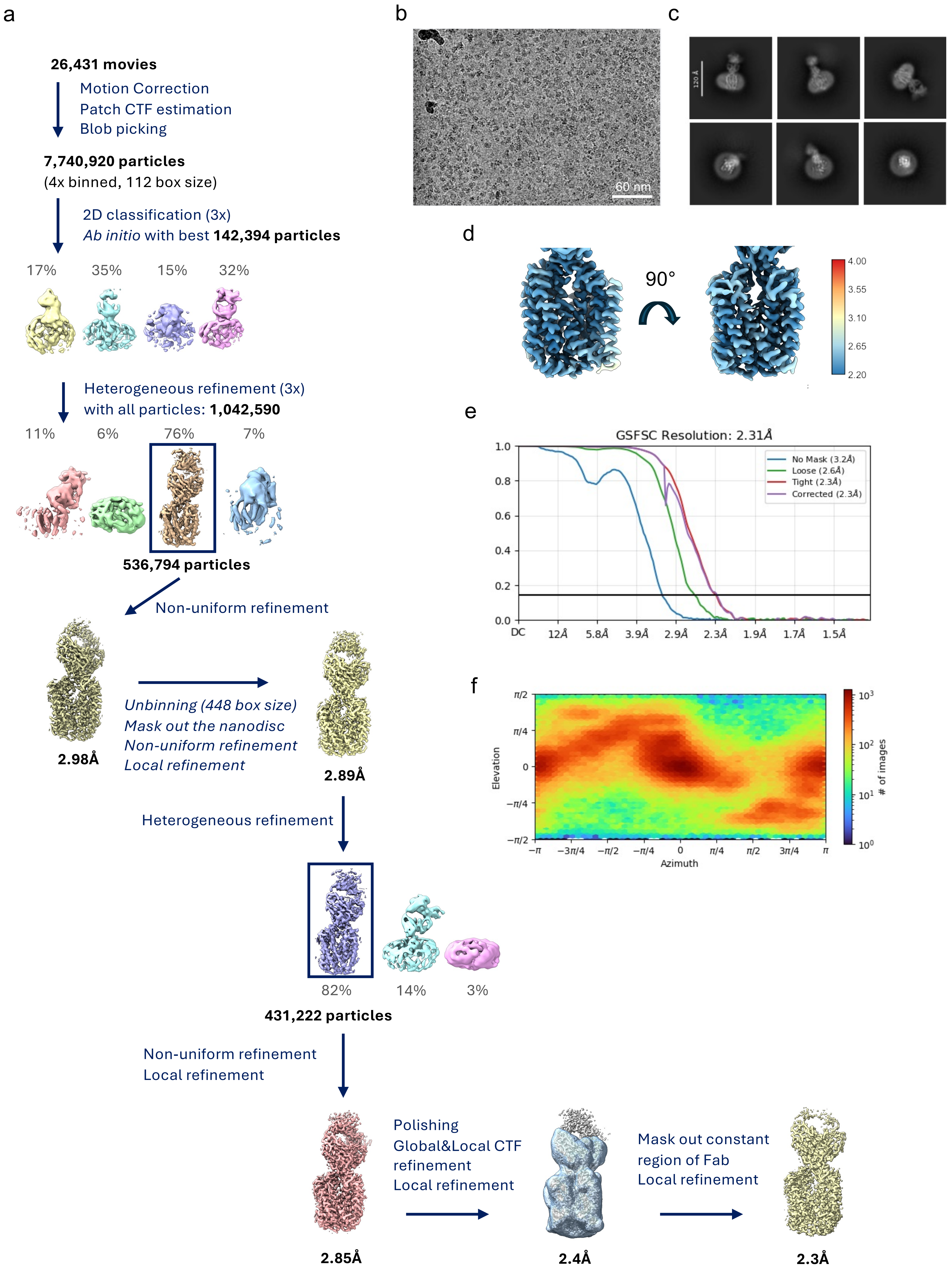

Supplementary Fig. 4. Cryo-EM analysis of hMATE1 in a complex with MATE1_Fab 6, elbow nanobody and metformin. **a** Flowchart presenting the pipeline of data processing in CryoSparc. **b** Representative micrograph of imaged sample. **c** Selected 2D classes. **d** Local resolution estimates of the final EM density map. **e** FSC curves of the final EM density maps. **f** Angular distribution of particles used in the final reconstruction.

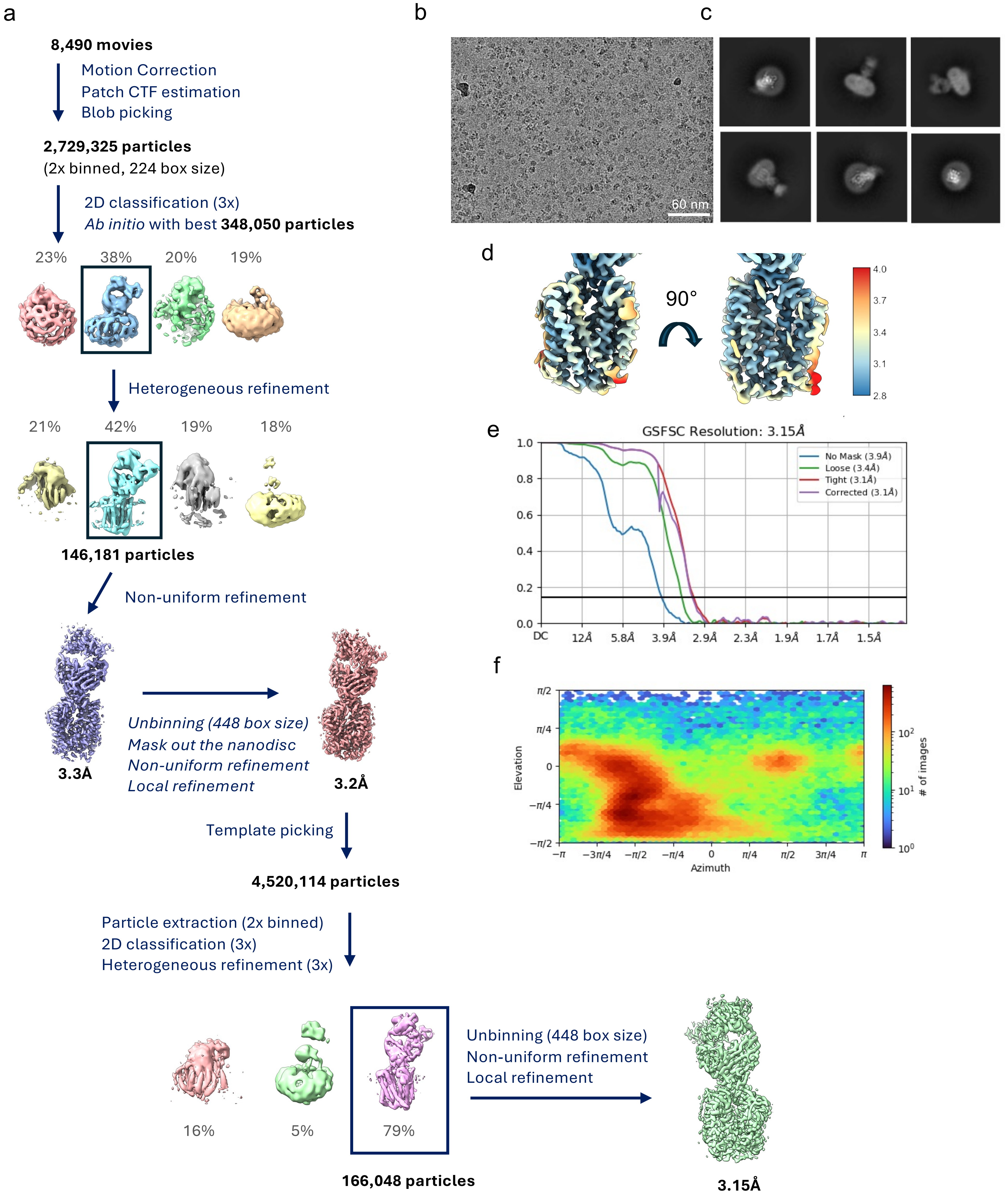

Supplementary Fig. 5. Cryo-EM analysis of hMATE1 in a complex with MATE1_Fab 6, elbow nanobody and MPP. **a** Flowchart presenting the pipeline of data processing in CryoSparc. **b** Representative micrograph of imaged sample. **c** Selected 2D classes. **d** Local resolution estimates of the final EM density map. **e** FSC curves of the final EM density maps. **f** Angular distribution of particles used in the final reconstruction.

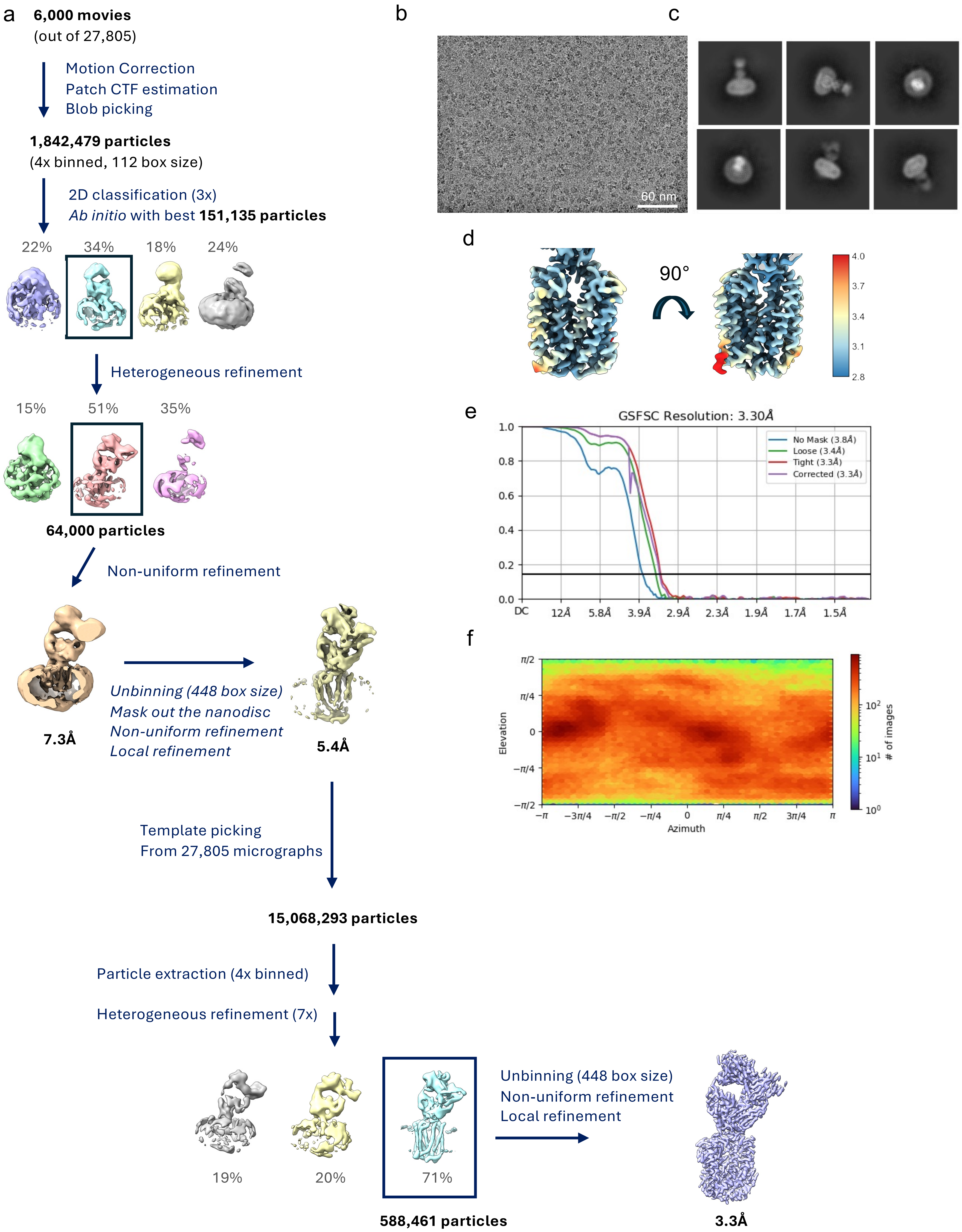

Supplementary Fig. 6. Cryo-EM analysis of hMATE1 in a complex with MATE1_Fab 6, elbow nanobody and cimetidine. **a** Flowchart presenting the pipeline of data processing in CryoSparc. **b** Representative micrograph of imaged sample. **c** Selected 2D classes. **d** Local resolution estimates of the final EM density map. **e** FSC curves of the final EM density maps. **f** Angular distribution of particles used in the final reconstruction.

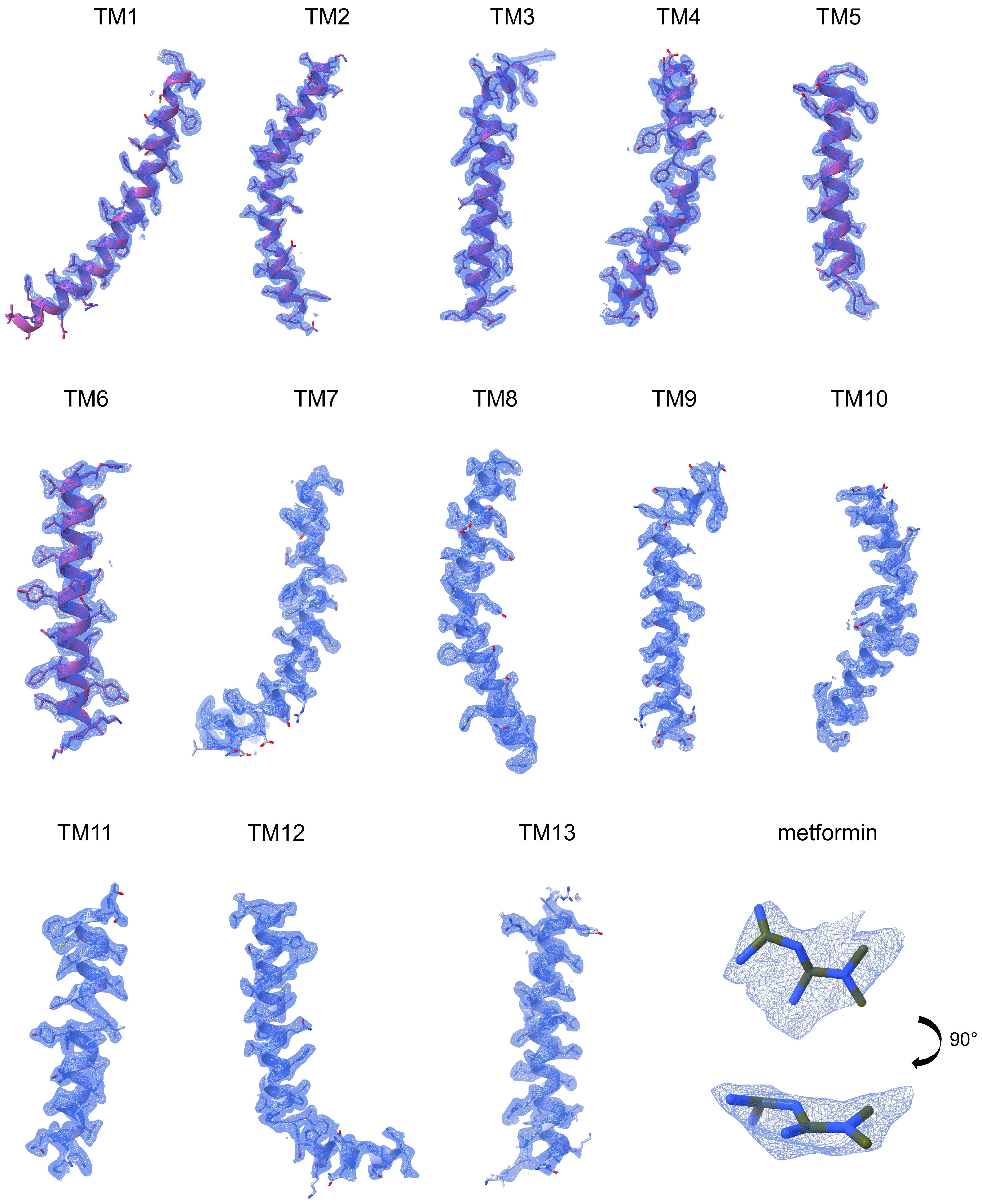

Supplementary Fig. 7. **a** Structures of individual transmembrane helices (TM) of hMATE1-MF with the cryo-EM density map shown within 2 Å of all atoms. **b** Fit of the metformin molecule into the cryo-EM density map shown from two different views.

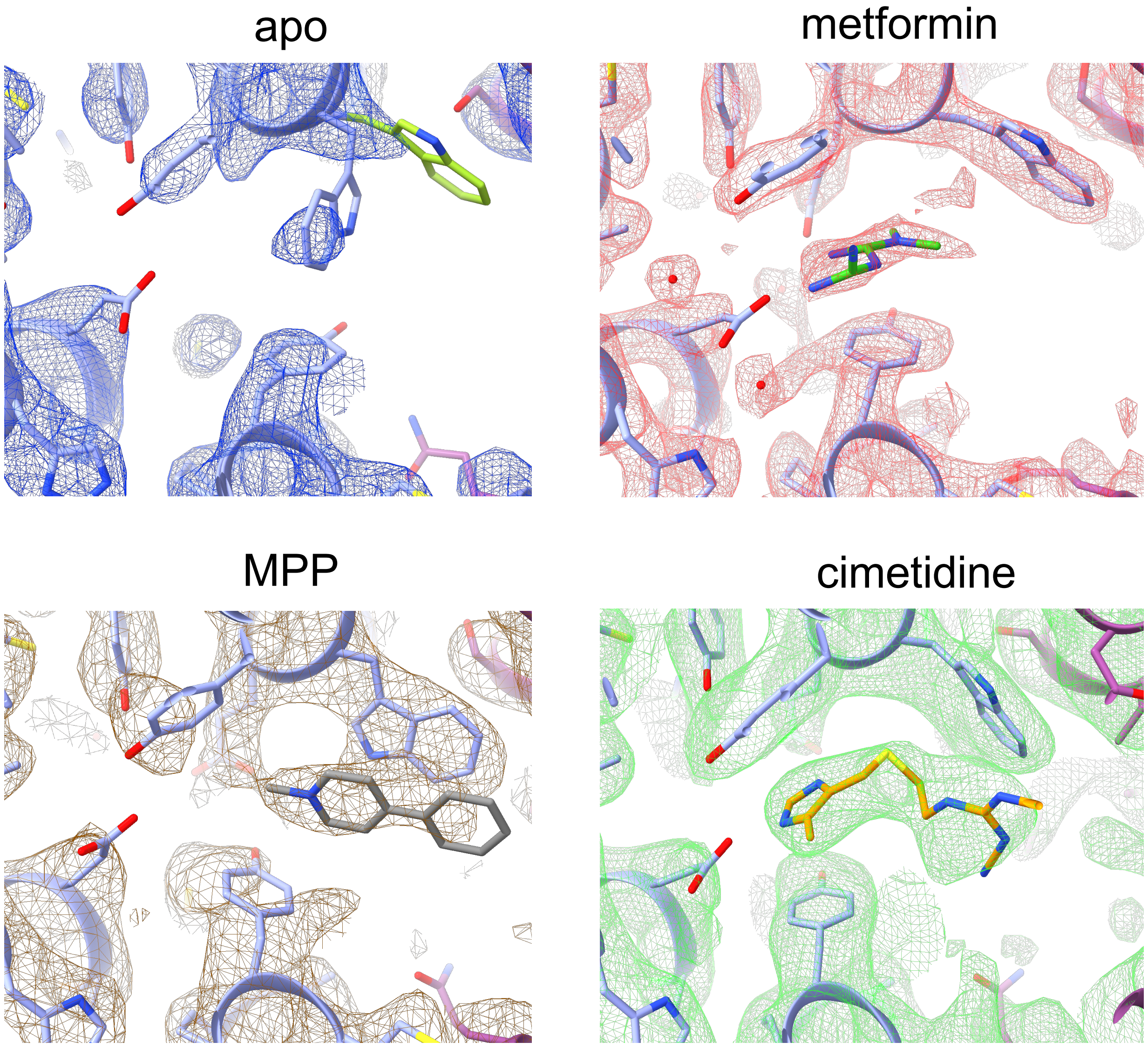

Supplementary Fig. 8. Cryo-EM map densities for the apo, metformin-bound, MPP-bound, and cimetidine-bound hMATE1 complexes shown at comparable thresholds and from identical views.

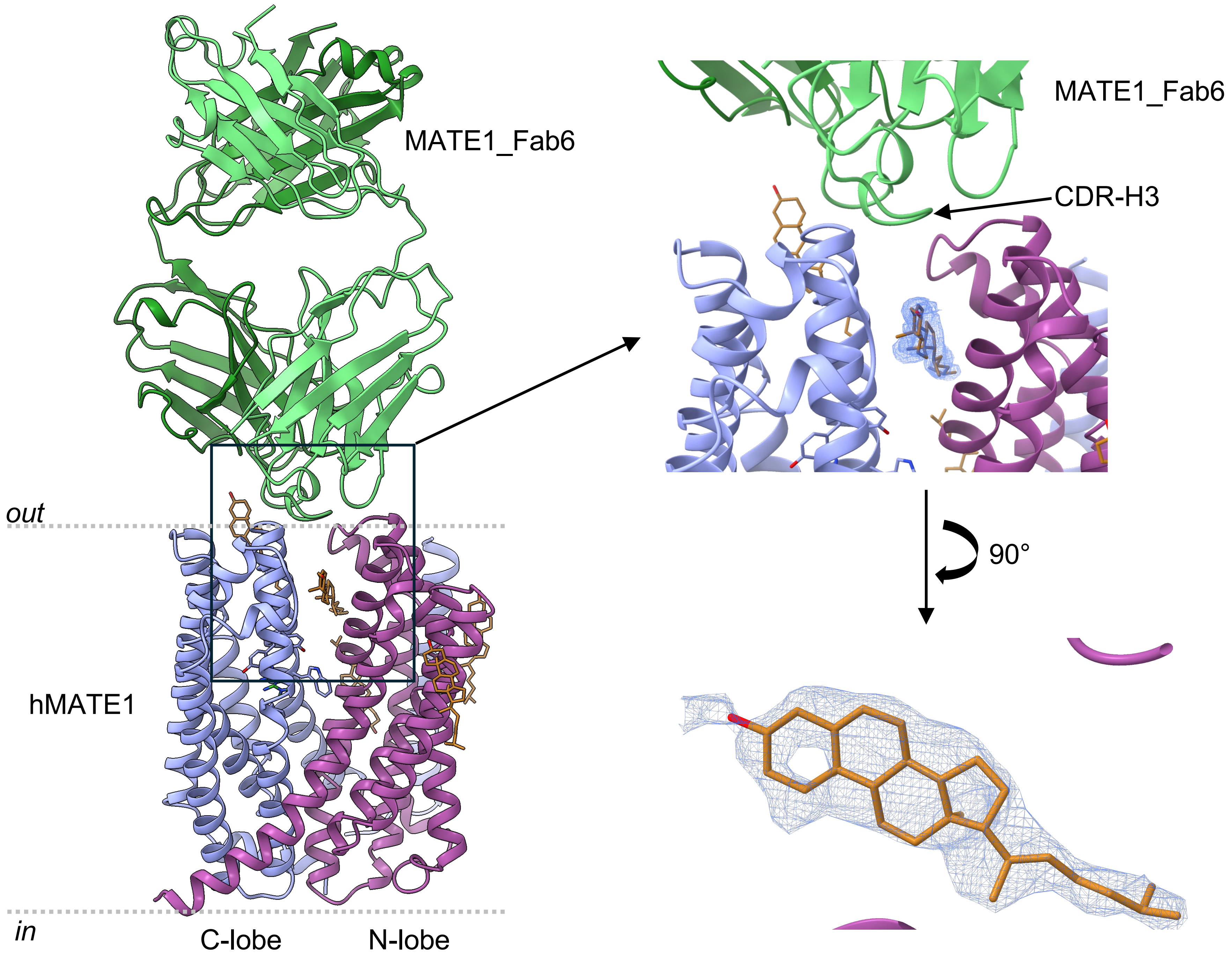

Supplementary Fig. 9. Density corresponding to cholesterol molecule was found in the central cleft between N- and C-lobe.

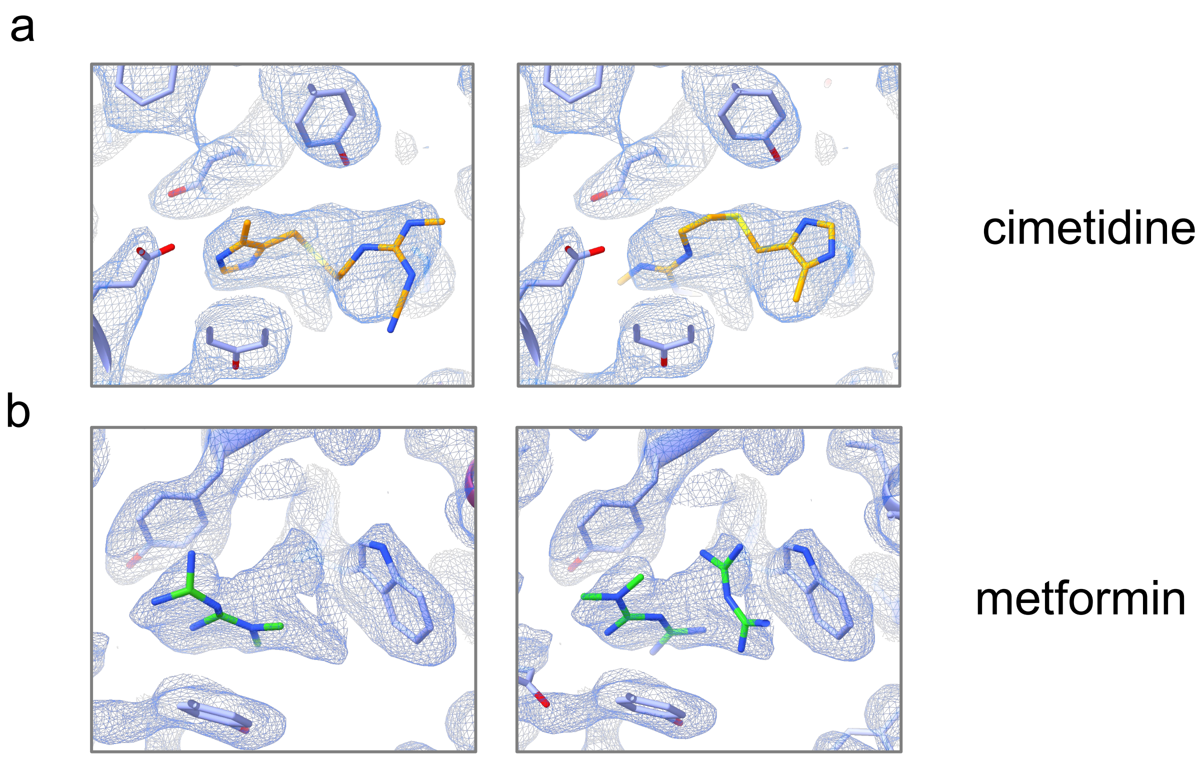

Supplementary Fig. 10. Alternative binding poses of **a** cimetidine and **b** metformin evaluated by MD simulations.

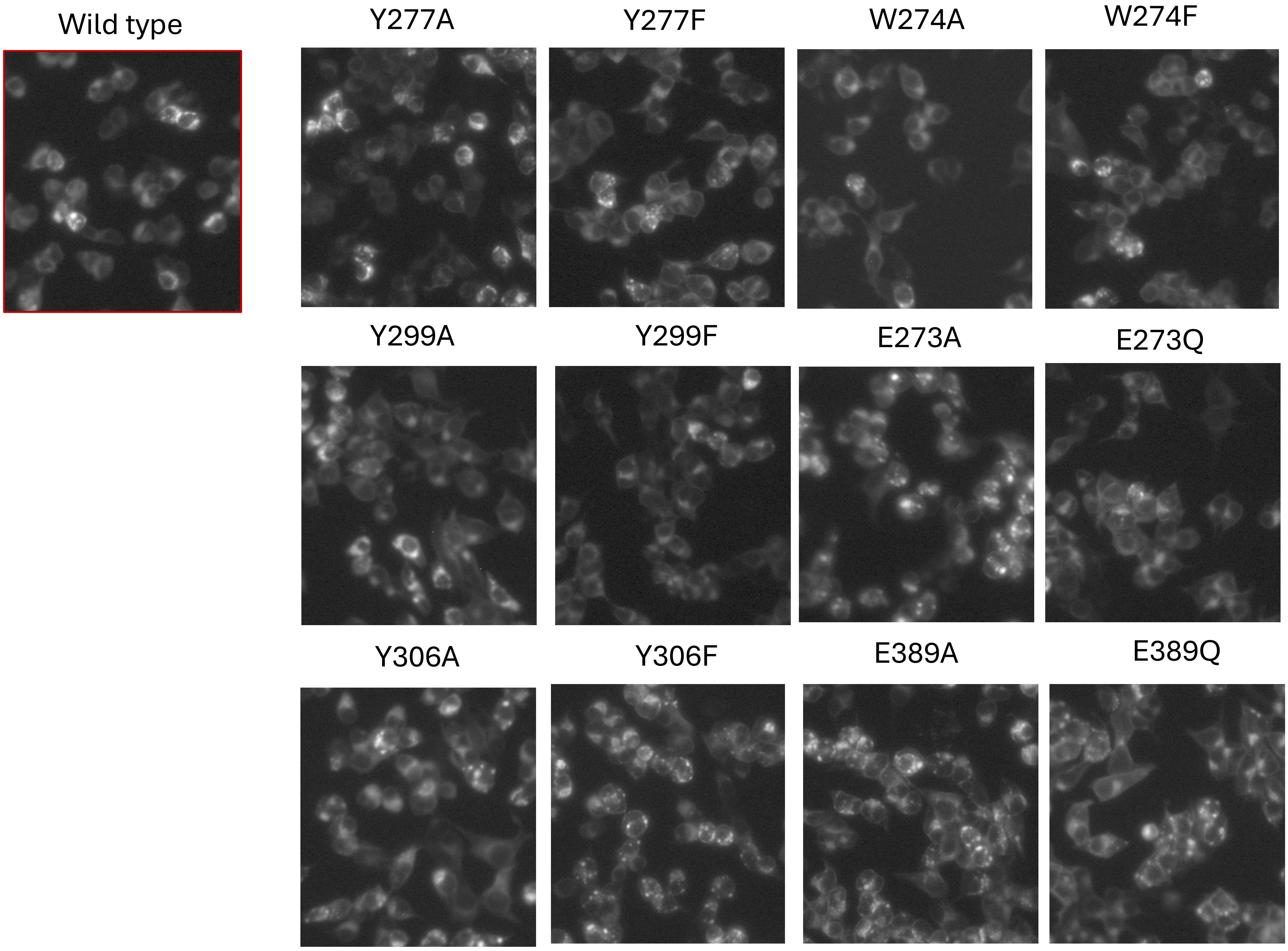

Supplementary Fig. 11. Fluorescent microscopic images showing membrane expression of hMATE1-eYFP mutants. eYFP signal shown as grayscale to emphasize the membrane expression level among different hMATE1 mutants.

a

0.3g cells (each mutant)
↓
Lysis and solubilization
↓
Extraction of total cellular membrane proteins
↓
Extraction of plasma membrane proteins
↓
SDS-PAGE gel
↓
Fluorescence detection (YFP signal) **b**
↓
Normalization to total protein loaded on gel
↓
Relative hMATE1 expression estimation (ImageJ) **c**

b

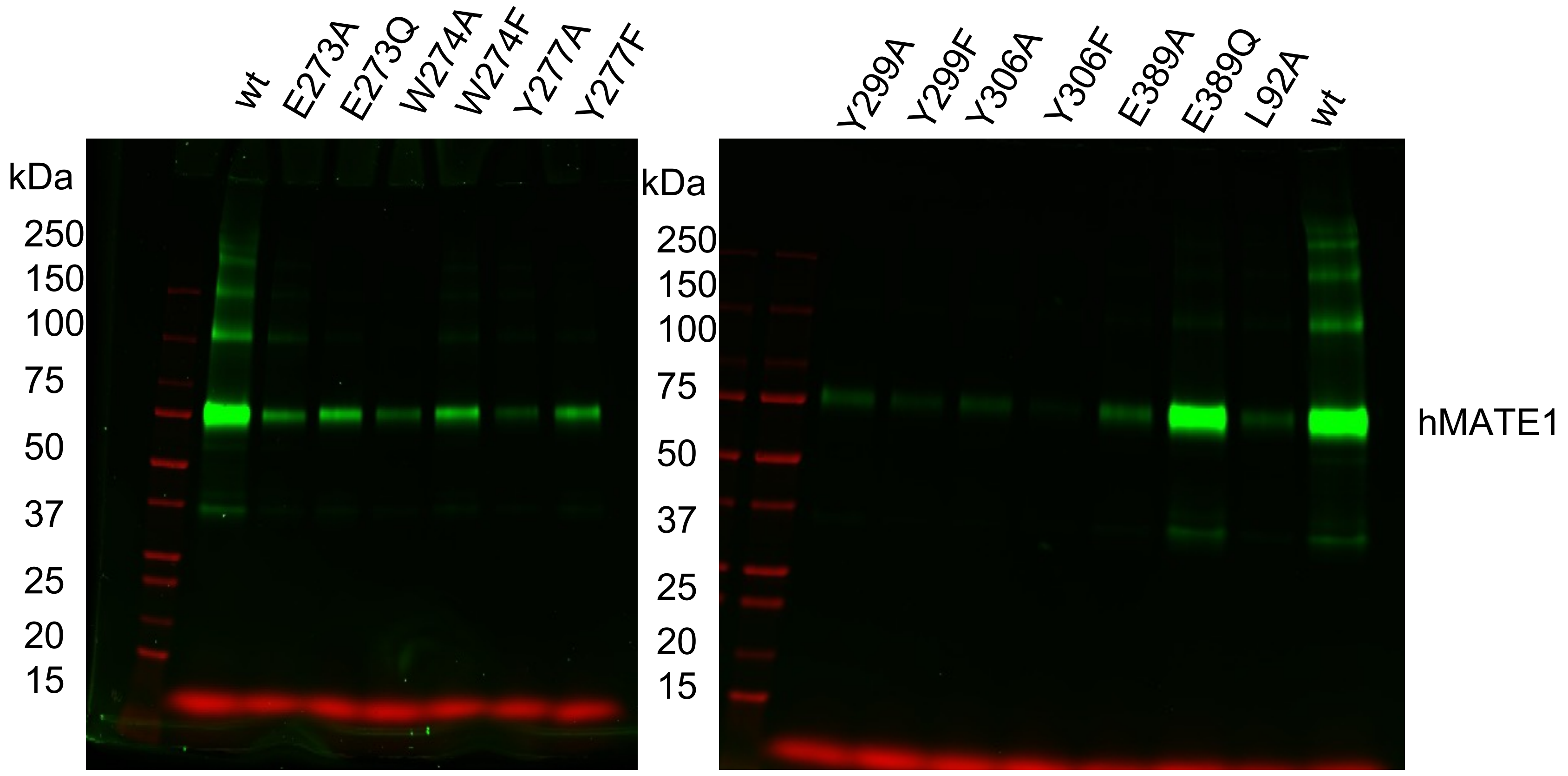

c

	Relative to wt
wt	1
E273A	0.47
E273Q	1.48
W274A	0.90
W274F	0.75
Y277A	0.58
Y277F	2.1

	Relative to wt
wt	1
Y299A	0.21
Y299F	0.14
Y306A	0.18
Y306F	0.16
E389A	0.59
E389Q	1.98
L92A	0.27

Supplementary Fig. 12. Membrane protein normalization for each mutant. **a** Protocol overview **b** Fluorescent gel with plasma membrane fraction of each mutant. **c** Relative expression of each mutant to the wild-type

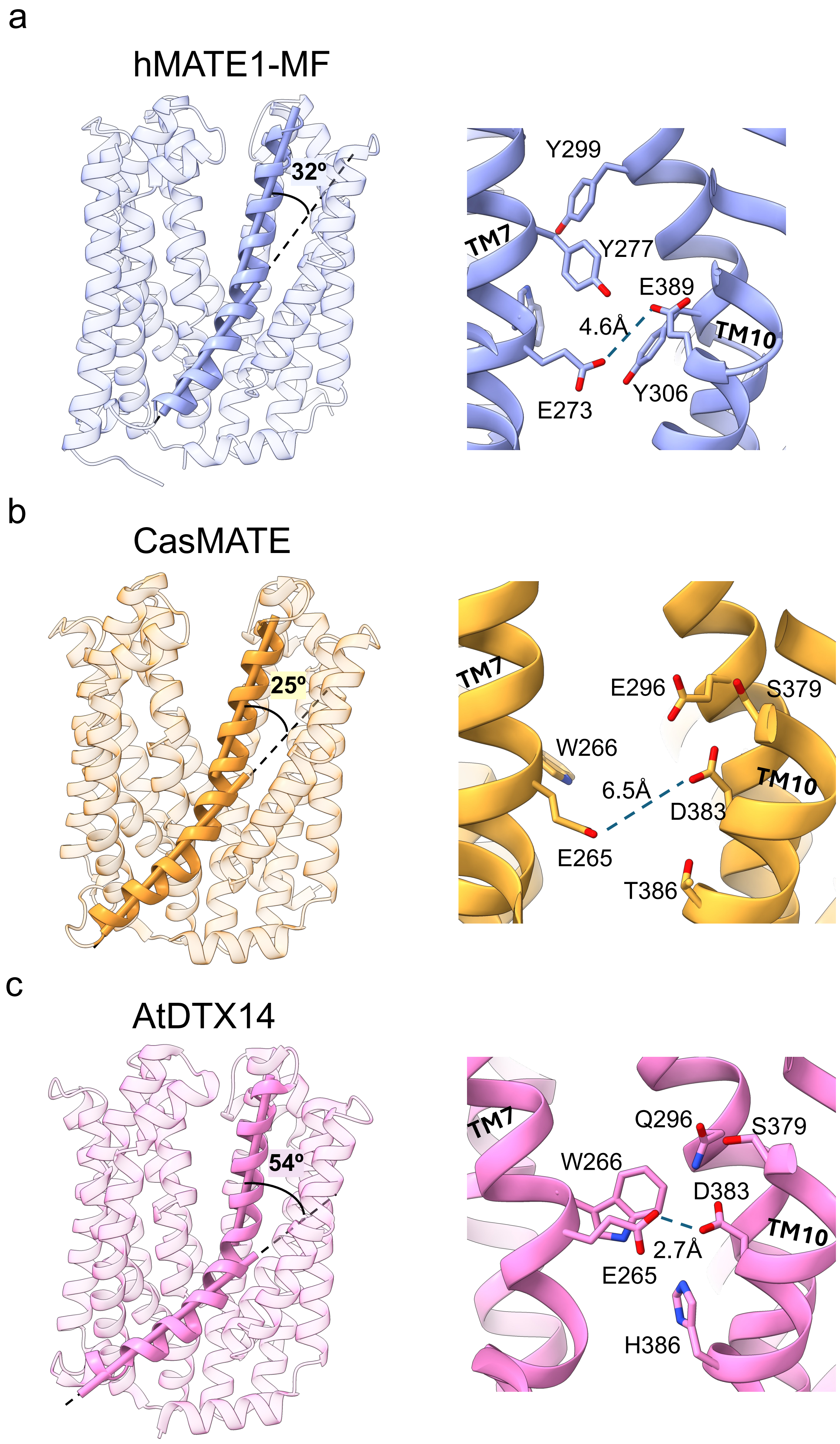

Supplementary Fig. 13. Comparison of eukaryotic MATE structures. **a** Left panel: Ribbon representation of the hMATE1 bound to metformin, showing the TM7 degree of bending. Right panel: Close-up view of the substrate binding pocket, displaying the distance between E273 and E389. Metformin is hidden for clarity. **b** Same as **a** for CasMATE. Right panel displays the distance between conserved E265 and D383. **c** Same as **b** for AtDTX14.

Supplementary Table 1. Cryo-EM data collection, refinement and validation statistics.

	hMATE1-CMT (EMDB-53489) (PDB-9R10)	hMATE1-MF (EMDB-53507) (PDB-9R1F)	hMATE1-MPP (EMDB-53506) (PDB-9R1E)	hMATE1-apo (EMDB-53508) (PDB-9R1G)
**Data collection and processing**				
Magnification	130,000	130,000	130,000	130,000
Voltage (kV)	300	300	300	300
Electron exposure (e–/Å^2)	51	42.5	51	53
Defocus range (μm)	-0.6 to -2.4	-0.6 to -2.4	-0.6 to -2.4	-0.6 to -2.4
Pixel size (Å)	0.65	0.65	0.65	0.65
Symmetry imposed	C1	C1	C1	C1
Initial particle images (no.)	15,068,293	7,740,920	2,729,325	2,606,923
Final particle images (no.)	588,461	431,222	166,048	327,752
Map resolution (Å)	3.30	2.31	3.15	2.95
FSC threshold	0.143	0.143	0.143	0.143
Map resolution range (Å)				
**Refinement**				
Initial model used (PDB code)	-	-	-	-
Model resolution (Å)	3.3	2.3	3.2	2.9
FSC threshold	0.143	0.143	0.143	0.143
Map sharpening *B* factor (Å^2)	-114	-125	-100	-102
Model composition				
Non-hydrogen atoms	7367	7204	7329	7245
Protein residues	924	919	924	919
Ligands	CMT :1<br>CLR:10	ME3 :1<br>CLR:5	MPP :1<br>CLR:9	-<br>CLR:8
B factors (Å^2)				
Protein	58.46	67.32	79.12	74.91
Ligand	77.61	63.62	90.14	51.57
R.m.s. deviations				
Bond lengths (Å)	0.004	0.002	0.002	0.003
Bond angles (°)	0.606	0.477	0.542	0.605
Validation				
MolProbity score	2.23	1.38	2.05	1.84
Clashscore	6.68	6.48	6.72	6.19
Poor rotamers (%)	4.51	3.80	3.10	2.08
Ramachandran plot				
Favored (%)	94.76	97.37	95.63	96.16
Allowed (%)	5.13	2.63	4.26	3.73
Disallowed (%)	0.00	0.00	0.11	0.11

Supplementary Table 2. Molecular Dynamics simulation system compositions.

System	Dimension (Å)	Number of atoms	Number of water molecules	Number of Cl^- atoms	Lipid composition
hMATE1-MF	100 × 100 × 100	98553	19382	10	POPC
hMATE1-MPP	100 × 100 × 100	98610	19381	11	POPC
hMATE1-CMT	100 × 100 × 100	98652	19381	11	POPC

POPC: 1-palmitoyl-2-oleoyl-sn-glycero-3-phosphocholine
