# Supplementary material for: Structural basis of drug recognition by human MATE1 transporter
Source: Nat Commun. 2025 Oct 27;16:9444. doi: 10.1038/s41467-025-64490-z (PMC12559748; doi:10.1038/s41467-025-64490-z)
Supplement: Supplementary file 2 — Description of Additional Supplementary Files [file 41467_2025_64490_MOESM2_ESM.pdf]

Description of Additional Supplementary Files

File name: Supplementary Data 1
Description: DNA primers used in this study.

File name: Supplementary Movie 1
Description: First 150 ns of MD simulation of hMATE1 with two metformin molecules in the binding pocket, one of which rapidly diffuses away.

File name: Supplementary Movie 2
Description: Representative 500 ns of MD simulation of hMATE1 with one metformin molecule, showing the dynamics of metformin within the binding pocket.

File name: Supplementary Movie 3
Description: First 100 ns of MD simulation of hMATE1 with the unstable modeled pose of cimetidine, which diffuses away.

File name: Supplementary Movie 4
Description: Representative 350 ns of MD simulation of hMATE1 with the stable pose of cimetidine, showing its dynamics within the binding pocket.

File name: Supplementary Movie 5
Description: Representative 350 ns of MD simulation of hMATE1 with MPP, showing its dynamics within the binding pocket.
