# Supplementary material for: Structural basis of drug recognition by human MATE1 transporter
Source: Nat Commun. 2025 Oct 27;16:9444. doi: 10.1038/s41467-025-64490-z (PMC12559748; doi:10.1038/s41467-025-64490-z)
Supplement: Supplementary file 9 — Reporting Summary [file 41467_2025_64490_MOESM9_ESM.pdf]

nature portfolio

Corresponding author(s): Kaspar P. Locher

Last updated by author(s): August 25 2025

Reporting Summary

Nature Portfolio wishes to improve the reproducibility of the work that we publish. This form provides structure for consistency and transparency in reporting. For further information on Nature Portfolio policies, see our Editorial Policies and the Editorial Policy Checklist.

Statistics

For all statistical analyses, confirm that the following items are present in the figure legend, table legend, main text, or Methods section.

n/a	Confirmed	
☐	☒	The exact sample size (*n*) for each experimental group/condition, given as a discrete number and unit of measurement
☐	☒	A statement on whether measurements were taken from distinct samples or whether the same sample was measured repeatedly
☒	☐	The statistical test(s) used AND whether they are one- or two-sided<br>*Only common tests should be described solely by name; describe more complex techniques in the Methods section.*
☒	☐	A description of all covariates tested
☒	☐	A description of any assumptions or corrections, such as tests of normality and adjustment for multiple comparisons
☐	☒	A full description of the statistical parameters including central tendency (e.g. means) or other basic estimates (e.g. regression coefficient) AND variation (e.g. standard deviation) or associated estimates of uncertainty (e.g. confidence intervals)
☒	☐	For null hypothesis testing, the test statistic (e.g. *F*, *t*, *r*) with confidence intervals, effect sizes, degrees of freedom and *P* value noted<br>*Give P values as exact values whenever suitable.*
☒	☐	For Bayesian analysis, information on the choice of priors and Markov chain Monte Carlo settings
☒	☐	For hierarchical and complex designs, identification of the appropriate level for tests and full reporting of outcomes
☒	☐	Estimates of effect sizes (e.g. Cohen's *d*, Pearson's *r*), indicating how they were calculated

Our web collection on statistics for biologists contains articles on many of the points above.

Software and code

Policy information about availability of computer code

Data collection	The Cryo-EM data were collected using EPU2 software (Thermo Fisher Scientific)
Data analysis	The Cryo-EM data were processed with CryoSparc v4.<br>Model building was performed with Coot 0.9.8.3, PHENIX 1.20.1-4487<br>Figures were prepared using UCSF Chimera X 1.7, GraphPad Prism v.10, Clustal Omega, JalView 2.11.2.6<br>For molecular dynamics, system assembly was performed using AmberTools (version 23.6). Simulations were performed using OpenMM (version 8.2.0). Trajectories were analyzed with VMD (version 1.9.4a57).

For manuscripts utilizing custom algorithms or software that are central to the research but not yet described in published literature, software must be made available to editors and reviewers. We strongly encourage code deposition in a community repository (e.g. GitHub). See the Nature Portfolio guidelines for submitting code & software for further information.

Data

Policy information about availability of data

All manuscripts must include a data availability statement. This statement should provide the following information, where applicable:

- Accession codes, unique identifiers, or web links for publicly available datasets
- A description of any restrictions on data availability
- For clinical datasets or third party data, please ensure that the statement adheres to our policy

Atomic coordinates of hMATE1-apo, hMATE1-MF, hMATE1-CMT and hMATE1-MPP were deposited in the Protein Data Bank under accession codes: 9R1G, 9R1F, 9R10 and 9R1E respectively. The corresponding cryo-EM maps were deposited in the Electron Microscopy Data Bank under EMD-53508, EMD-53507, EMD-53489, EMD-53506. Data related to molecular dynamics simulations are available at Zenodo: https://doi.org/10.5281/zenodo.15836460.

Research involving human participants, their data, or biological material

Policy information about studies with human participants or human data. See also policy information about sex, gender (identity/presentation), and sexual orientation and race, ethnicity and racism.

Reporting on sex and gender	not applicable
Reporting on race, ethnicity, or other socially relevant groupings	not applicable
Population characteristics	not applicable
Recruitment	not applicable
Ethics oversight	not applicable

Note that full information on the approval of the study protocol must also be provided in the manuscript.

Field-specific reporting

Please select the one below that is the best fit for your research. If you are not sure, read the appropriate sections before making your selection.

☒ Life sciences ☐ Behavioural & social sciences ☐ Ecological, evolutionary & environmental sciences

For a reference copy of the document with all sections, see nature.com/documents/nr-reporting-summary-flat.pdf

Life sciences study design

All studies must disclose on these points even when the disclosure is negative.

Sample size	Cell based transport assays were performed wth N=3 biological replicates
Data exclusions	Data points compromised by technical errors were omitted from the analysis
Replication	Replication is part of the single particle cryo-EM data processing, whereas cell-based transport assay is reproducible
Randomization	not applicable for structural study and cell-based transport assay
Blinding	not applicable for structural study and cell-based transport assay

Reporting for specific materials, systems and methods

We require information from authors about some types of materials, experimental systems and methods used in many studies. Here, indicate whether each material, system or method listed is relevant to your study. If you are not sure if a list item applies to your research, read the appropriate section before selecting a response.

Materials & experimental systems

n/a	Involved in the study	
☐	☒	Antibodies
☐	☒	Eukaryotic cell lines
☒	☐	Palaeontology and archaeology
☒	☐	Animals and other organisms
☒	☐	Clinical data
☒	☐	Dual use research of concern
☒	☐	Plants

Methods

n/a	Involved in the study	
☒	☐	ChIP-seq
☒	☐	Flow cytometry
☒	☐	MRI-based neuroimaging

Antibodies

Antibodies used: MATE1_Fab3 and MATE1_Fab6 were produced in-house from a synthetic library (as described in methods)

Validation: Biophysical and biochemical characterization of the Fabs is described in the results section

Eukaryotic cell lines

Policy information about cell lines and Sex and Gender in Research

Cell line source(s): Flp-In™ T-REx™ 293 Cell Line and HEK293T cells (Thermo Fisher Scientific)

Authentication: No authentication was performed

Mycoplasma contamination: No contamination tests were performed

Commonly misidentified lines (See ICLAC register): Not applicable

Plants

Seed stocks: Not applicable

Novel plant genotypes: Not applicable

Authentication: Not applicable
